# Development of a Nomogram to Classify In-Hospital Atrial Fibrillation Among Patients Hospitalized With Acute Myocardial Infarction: A Retrospective Case–Control Study

**DOI:** 10.31083/RCM45539

**Published:** 2025-11-27

**Authors:** Geng Yang, Long Feng, Yilin Pan, Mankun Xin, Wenwen Duan, Decheng Chen, Muhammad Taimoor Nasir, Shijie Yang, Xiaonan He

**Affiliations:** ^1^Department of Emergency, Beijing Anzhen Hospital, Capital Medical University, 100029 Beijing, China; ^2^Department of Clinical Medicine, Capital Medical University, 100029 Beijing, China

**Keywords:** acute myocardial infarction, atrial fibrillation, risk factors, prediction model

## Abstract

**Background:**

Previous studies on acute myocardial infarction (AMI) complicated by atrial fibrillation (AF) have mainly focused on anatomy or underlying disease state, and its prognostic predictors have not been fully explored. Therefore, there is a need for an effective prognosis model for patients with AMI-AF.

**Methods:**

We retrospectively selected 126 patients with acute myocardial infarction complicated with AF hospitalized in Beijing Anzhen Hospital from January 2020 to December 2024 as the case group, and 1719 patients without AF as the control group. The clinical characteristics and laboratory test results of the two groups were compared to determine independent risk factors for AF in patients with acute myocardial infarction. The predictive performance of the model was evaluated by plotting Receiver Operating Characteristic (ROC) for each independent predictor. For the combined model, we used R software to build pattern plots, calibration plots, and Decision Curve Analysis (DCA) based on a multivariate logistic regression model.

**Results:**

Multivariate Logistic regression analysis showed that older age (Odds Ratio (OR) = 1.067, 95% CI: 1.044–1.092), longer hospitalization days (OR = 1.039, 95% CI: 1.013–1.066). The AUCs for age, hospitalization days, history of coronary heart disease, heart rate, International Normalized Ratio (INR), Hemoglobin, and mean platelet volume were 0.721, 0.663, 0.577, 0.614, 0.688, 0.438, and 0.607. The AUC of nomogram model for predicting AF in AMI patients was 0.833 (95% CI: 0.796–0.870, *p* < 0.001), the sensitivity was 0.817, and the specificity was 0.726. The nomogram model indicated a clinical net benefit when the predicted risk threshold exceeded 0.06.

**Conclusions:**

Multivariable prediction model has good prediction effect. The variables in this nomogram model are easily obtained in clinical practice and can provide reference for individualized prediction of AF in AMI patients.

## 1. Introduction

Acute myocardial infarction (AMI) is a critical cardiovascular emergency caused by acute coronary artery occlusion leading to myocardial ischemic necrosis. Its morbidity and mortality remain elevated globally [1]. Although advances in percutaneous coronary intervention (PCI) and medical treatment have significantly improved the short-term prognosis of AMI, its complications (such as arrhythmia and heart failure) remain a major challenge affecting patient’s quality of life and long-term prognosis [2]. Among them, atrial fibrillation (AF) is a common complication of AMI, with an incidence rate of about 10% to 20%, and is closely related to patient mortality, stroke risk and cardiac function deterioration [3]. Studies have shown that the in-hospital mortality rate among patients with AMI and AF is 2–3 times higher than that of patients with AMI alone, and the long-term prognosis is worse [4].

The high incidence of AF in AMI patients is closely related to the complex pathophysiological mechanisms. Myocardial injury and ventricular remodeling after AMI disrupt atrial electrophysiology and promote AF [5]. Systemic inflammation, oxidative stress, and neurohormonal activation further contribute to atrial fibrosis and electrical instability [6]. In turn, AF exacerbates myocardial ischemia, reduces cardiac output, and increases thromboembolic risk [7]. Although previous studies have identified traditional risk factors such as age, left ventricular dysfunction, hypertension, and diabetes, these indicators rely mostly on anatomy or underlying disease status, and may not fully reflect the dynamic pathological process of AF in AMI patients [8]. Based on this, this study fully integrates hematological indicators and parameters such as cardiac ultrasound, and through large-scale clinical data analysis which aims to reveal the risk factors of AF in AMI patients and establish a risk prediction model, providing evidence-based basis for early identification of high-risk populations and the development of personalized intervention strategies.

## 2. Methods

### 2.1 Case Selection

**Fig. 1.**
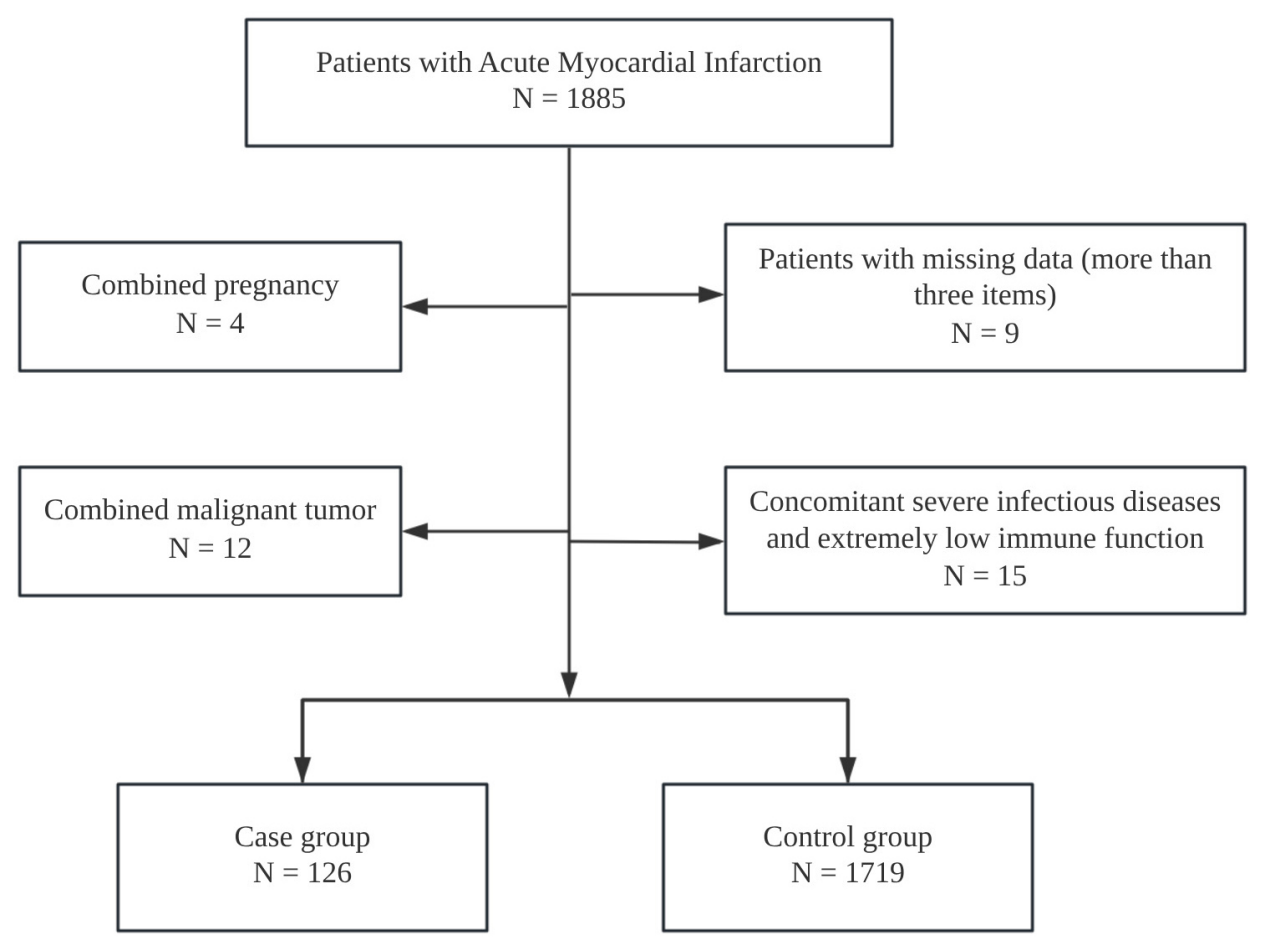
**A consort type diagram of whole patients with acute myocardial infarction**.

We selected 126 patients hospitalized with AMI complicated by AF in Beijing Anzhen Hospital Affiliated to Capital Medical University from January 2020 to December 2024 as the case group, and 1719 in patients with AMI without AF as the control group. The inclusion criteria were: age more than 18 years; meet the diagnostic criteria of AMI [9]; meet the diagnostic criteria of AF [10]. Exclusion criteria are: incomplete clinical data such as vital signs; pregnancy; concomitant severe infectious diseases and extremely low immune function. In total, 1845 participants were included in the final analysis (Fig. 1). This study was reviewed by the Ethics Committee of Beijing Anzhen Hospital Affiliated to Capital Medical University (Approval No. 2024-05).

This was a retrospective case-control study, and the sample size was estimated based on the “Events per Variable” principle. Based on previous research recommendations, at least 10 positive events are required for each predictor variable. This study ultimately included 7 independent risk factors, so at least 70 patients with AMI complicated by AF were needed. 126 cases were actually included, and there were sufficient concurrent events to meet the model construction needs.

### 2.2 Clinical Data Collection

All members of the investigation team have been trained on unified standards. Trained staff explained the purpose, implementation process, and benefits of this study to eligible research subjects within 24 hours of admission, and obtained their consent. Trained research staff abstracted data from the hospital’s electronic medical records and collected information about patients who agreed to participate in the study according to the designed data questionnaire. All information was checked by two people and entered into an Excel sheet. Atrial fibrillation was ascertained by 12-lead Electrocardiogram (ECG) or continuous telemetry interpreted by board-certified cardiologists during the index hospitalization, from emergency department triage to discharge. AF status therefore reflects AF present at admission or documented at any time before discharge. Predictors were abstracted at or near admission (Emergency Department (ED) triage/first-day vitals and initial laboratory tests, generally within 24 hours). For patients with AF on admission, the nearest available pre-AF measurements were used when possible.

Detailed medical history was collected for all patients (diagnosis complied with relevant guideline standards). Clinical characteristics collected included: Age, gender, body mass index (BMI), length of stay; Medical history: hypertension [11], diabetes [12], coronary heart disease [13], old myocardial infarction [14], history of PCI, coronary artery bypass grafting (CABG), stroke [15], peripheral vascular disease [16], history of smoking and alcohol consumption; Clinical complications included: Killip ≥Grade III [9], Heart failure [17], Lung infection [18], Renal dysfunction [19], Hepatic dysfunction [20], Hypoproteinemia [21], Hyperuricemia [22]; Patient vital signs on admission included: systolic blood pressure, diastolic blood pressure, heart rate; Cardiac ultrasound: ejection fraction, pericardial effusion, aortic valve regurgitation; Patient laboratory parameters (using the first test results on admission) included: serum triglyceride (TG), total cholesterol (TC), high-density lipoprotein cholesterol (HDL-C), low-density lipoprotein cholesterol (LDL-C), Activated Partial Thromboplastin Time (APTT), International Normalized Ratio (INR), fibrin degradation products (FDP), D-dimer (DD), white blood cells (WBC), Hemoglobin (Hb), platelets (PLT), Mean Platelet Volume (MPV), Neutrophils Percentage (NEUT%), Neutrophils Count (NEUT).

### 2.3 Statistical Methods

The measurement data were subjected to Shapiro-Wilk normality test. The measurement data conforming to the normal distribution were expressed as mean ± standard deviation (SD), and the comparison between groups was carried out using the two-independent-sample *t*-test; the measurement data non-normal distribution was expressed as median and quartile [*M* (*Q*_1_, *Q*_3_)], and the comparison between groups was carried out using the Mann-Whitney U test. Counting data are expressed as frequency (rate) [*n* (%)], and χ^2^ test is used for comparison between groups. We performed multivariable logistic regression analysis to identify independent risk factors. Based on these results, we constructed a risk prediction model, with *p* < 0.05 as the difference being statistically significant. The predictive performance of the model was evaluated by plotting each independent predictor and the Area under the ROC for joint diagnosis. R version 4.4.3 (R Core Team, Auckland, New Zealand) was used to plot nomograms, calibration plots and Decision Curve Analysis (DCA) based on the multi-factor Logistic regression model to evaluate the clinical efficacy of the nomogram model, other statistical analyses using SPSS software (version 25.0, IBM Corp., Armonk, NY, USA).

## 3. Results

### 3.1 General Clinical Data and Single Factor Analysis of Patients

Univariate analysis revealed significant differences between the case and control groups for the following variables: age, length of stay, hypertension, diabetes, coronary heart disease, old myocardial infarction, stroke, peripheral vascular disease, Killip ≥III, heart failure, lung infection, renal insufficiency, hypoproteinemia, hyperuricemia, diastolic blood pressure, heart rate, ejection fraction, pericardial effusion, aortic valve regurgitation, TG, TC, LDL-C, INR, FDP, Hb, PLT, MPV, NEUT%, NEUT (*p* < 0.05). There was no significant difference in gender distribution and BMI between the case group and the control group (*p* > 0.05). Compared with the control group, the proportion of patients with history of PCI and CABG surgery in the case group was higher than that in the control group, but the difference was not statistically significant (*p* > 0.05). The proportion of patients with history of smoking and drinking in the case group was lower than that in the control group, but the difference was not statistically significant (*p* > 0.05). Compared with the control group, the proportion of patients in the case group who had concurrent liver dysfunction during hospitalization was higher than that in the control group, but the difference was not statistically significant (*p* > 0.05). The proportion of patients with a history of PCI or CABG was higher in the case group than in the control group, but this difference was not statistically significant (*p* > 0.05). Compared with the control group, the levels of HDL-C, APTT, DD and WBC in laboratory parameters of patients in the case group were higher than those in the control group, but the difference was not statistically significant (*p* > 0.05) (Table 1).

**Table 1.** **Comparison of general clinical data and biochemical indicators between the two groups of patients**.

Characteristic		Case Group (*n* = 126)	Control Group (*n* = 1719)	χ^2^/*t*/*Z*	*p*-value
Clinical characteristics					
	Age, mean ± SD, years	68.60 ± 9.42	59.35 ± 12.08	10.410	<0.001
	Male, n (%)	96 (76.19)	1383 (80.45)	1.087	0.297
	Female, n (%)	30 (23.81)	336 (19.55)	1.087	0.297
	BMI, mean ± SD	25.35 ± 3.54	25.99 ± 3.71	1.897	0.058
	Hospitalization days, Median (IQR)	8 (5, 12)	5 (3, 8)	–3.695	<0.001
Medical history, n (%)					
	Hypertension	92 (73.02)	1013 (58.93)	9.120	0.003
	Diabetes	63 (50.00)	662 (38.51)	6.023	0.014
	Coronary heart disease	81 (64.29)	841 (48.92)	10.476	0.001
	Old myocardial infarction	23 (18.25)	186 (10.82)	5.740	0.017
	PCI surgery	32 (25.40)	313 (18.21)	3.532	0.060
	CABG surgery	9 (7.14)	69 (4.01)	2.118	0.146
	Stroke	24 (19.05)	193 (11.23)	6.185	0.013
	Peripheral vascular disease	6 (4.76)	28 (1.63)	4.756	0.029
	Smoking status	39 (30.95)	649 (37.75)	2.041	0.153
	Alcohol consumption	20 (15.87)	351 (20.42)	1.240	0.265
Clinical complication, n (%)					
	Killip ≥Grade III	18 (14.29)	77 (4.48)	21.151	<0.001
	Heart failure	28 (22.22)	115 (6.69)	37.469	<0.001
	Lung infection	25 (19.84)	75 (4.36)	51.887	<0.001
	Renal dysfunction	30 (23.81)	143 (8.32)	31.354	<0.001
	Hepatic dysfunction	13 (10.32)	125 (7.27)	1.164	0.281
	Hypoproteinemia	29 (23.02)	137 (7.97)	30.647	<0.001
	Hyperuricemia	12 (9.52)	73 (4.25)	6.287	0.012
Vital signs					
	Systolic blood pressure, mean ± SD	125 ± 20	126 ± 19	–0.568	0.570
	Diastolic blood pressure, mean ± SD	74 ± 12	76 ± 12	–1.806	0.071
	Heart rate, Median (IQR)	84 (78, 90)	79 (73, 85)	–4.002	<0.001
Cardiac ultrasound					
	Ejection fraction, Median (IQR), %	46 (36, 56)	55 (45, 61)	5.931	<0.001
	Pericardial effusion, n (%)	15 (11.90)	103 (5.99)	5.904	0.015
	Aortic valve regurgitation, n (%)	67 (53.17)	513 (29.84)	28.577	<0.001
Laboratory parameters					
	TG, Median (IQR), mmol/L	1.24 (0.89, 1.59)	1.51 (1.11, 2.15)	7.051	<0.001
	TC, Median (IQR), mmol/L	3.59 (2.98, 4.27)	4.08 (3.40, 4.85)	4.160	<0.001
	HDL-C, Median (IQR), mmol/L	1.04 (0.87, 1.21)	0.99 (0.84, 1.17)	–1.360	0.174
	LDL-C, Median (IQR), mmol/L	1.95 (1.49, 2.70)	2.35 (1.75, 3.06)	3.588	<0.001
	APTT, Median (IQR), Sec	32.65 (29.33, 38.45)	31.00 (28.80, 34.00)	–1.474	0.141
	INR, Median (IQR)	1.07 (1.02, 1.21)	1.02 (0.97, 1.07)	–4.907	<0.001
	FDP, Median (IQR), ug/mL	1.40 (0.78, 4.39)	0.90 (0.60, 1.70)	–2.013	0.044
	DD, Median (IQR), µg/L	161.00 (82.25, 567.25)	108.00 (63.00, 208.00)	–1.787	0.074
	WBC, Median (IQR), ×10^9^/L	7.71 (6.18, 10.53)	7.41 (6.90, 34.00)	–1.972	0.051
	HB, Median (IQR), g/L	133 (117, 151)	140 (126, 151)	2.440	0.016
	PLT, Median (IQR), ×10^9^/L	196 (162, 241)	223 (185, 267)	4.007	<0.001
	MPV, mean ± SD, fL	10.25 ± 1.06	9.85 ± 1.06	–4.060	<0.001
	NEUT%, mean ± SD, %	74.91 ± 10.99	70.43 ± 9.99	–4.819	<0.001
	NEUT, Median (IQR), ×10^9^/L	5.83 (4.19, 8.25)	5.12 (3.96, 6.89)	–3.756	<0.001

BMI, body mass index; PCI, percutaneous coronary intervention; CABG, coronary artery bypass grafting; TG, triglyceride; TC, total cholesterol; HDL-C, high-density lipoprotein cholesterol; LDL-C, low-density lipoprotein cholesterol; APTT, Activated Partial Thromboplastin Time; INR, International Normalized Ratio; FDP, fibrin degradation products; DD, D-dimer; WBC, white blood cells; HB, Hemoglobin; PLT, platelets; MPV, Mean Platelet Volume; NEUT%, Neutrophils Percentage; NEUT, Neutrophils Count.

### 3.2 Multivariate Logistic Analysis of Risk Factors for AF in Patients With AMI

Whether AMI patients had AF was used as the dependent variable, and items with statistically significant differences within single factors were used as the independent variable. The difference variables screened by the above single-factor analysis were included in the Logistic model for multivariate analysis. The results showed that older age, longer hospital stay, previous history of coronary heart disease, faster heart rate, higher INR level, lower Hb level, and larger mean platelet volume were all independent risk factors for AF in AMI patients (*p* < 0.05) (Table 2).

**Table 2.** **Multivariate logistic regression analysis of the incidence of AF in AMI patients**.

Characteristic		*b*	S_b_	Wald χ^2^	*OR* (95% *CI*)	*p*-value
Clinical characteristics						
	Age, years	0.065	0.011	33.008	1.067 (1.044, 1.092)	<0.001
	Hospitalization days	0.038	0.013	8.529	1.039 (1.013, 1.066)	0.003
Medical history						
	Hypertension	0.417	0.236	3.110	1.517 (0.955, 2.41)	0.078
	Diabetes	0.317	0.213	2.220	1.373 (0.905, 2.084)	0.136
	Coronary heart disease	0.471	0.219	4.620	1.602 (1.042, 2.462)	0.032
	Old myocardial infarction	–0.187	0.304	0.379	0.829 (0.457, 1.506)	0.538
	Stroke	0.043	0.268	0.025	1.044 (0.617, 1.766)	0.873
	Peripheral vascular disease	–0.273	0.651	0.176	0.761 (0.212, 2.727)	0.675
Clinical complication						
	Killip ≥Grade III	0.063	0.372	0.029	1.065 (0.514, 2.21)	0.865
	Heart failure	0.538	0.300	3.212	1.712 (0.951, 3.081)	0.073
	Lung infection	0.436	0.358	1.487	1.547 (0.767, 3.118)	0.223
	Renal dysfunction	0.190	0.312	0.371	1.209 (0.656, 2.228)	0.542
	Hypoproteinemia	0.397	0.309	1.651	1.487 (0.812, 2.724)	0.199
	Hyperuricemia	0.577	0.392	2.166	1.781 (0.826, 3.842)	0.141
Vital signs						
	Diastolic blood pressure	0.005	0.010	0.301	1.005 (0.987, 1.024)	0.583
	Heart rate	0.021	0.007	8.218	1.021 (1.007, 1.035)	0.004
Cardiac ultrasound						
	Ejection fraction, %	–0.014	0.009	2.354	0.986 (0.968, 1.004)	0.125
	Pericardial effusion	–0.172	0.357	0.232	0.842 (0.418, 1.695)	0.630
	Aortic valve regurgitation	0.344	0.214	2.574	1.411 (0.927, 2.147)	0.109
Laboratory parameters						
	TG, mmol/L	–0.252	0.178	2.007	0.777 (0.549, 1.101)	0.157
	TC, mmol/L	–0.101	0.369	0.074	0.904 (0.439, 1.862)	0.785
	LDL-C, mmol/L	–0.062	0.410	0.023	0.94 (0.421, 2.101)	0.881
	INR	0.716	0.312	5.275	2.047 (1.111, 3.773)	0.022
	FDP, ug/mL	–0.026	0.014	3.373	0.975 (0.948, 1.002)	0.066
	HB, g/L	0.017	0.005	10.375	1.018 (1.007, 1.028)	0.001
	PLT, ×10^9^/L	–0.001	0.002	0.384	0.999 (0.996, 1.002)	0.535
	MPV, fL	0.205	0.100	4.185	1.227 (1.009, 1.493)	0.041
	NEUT%, %	0.022	0.015	2.260	1.022 (0.993, 1.052)	0.133
	NEUT, ×10^9^/L	0.012	0.046	0.075	1.013 (0.926, 1.107)	0.785

### 3.3 Constructing a Nomogram Model to Predict AF in AMI Patients

**Fig. 2.**
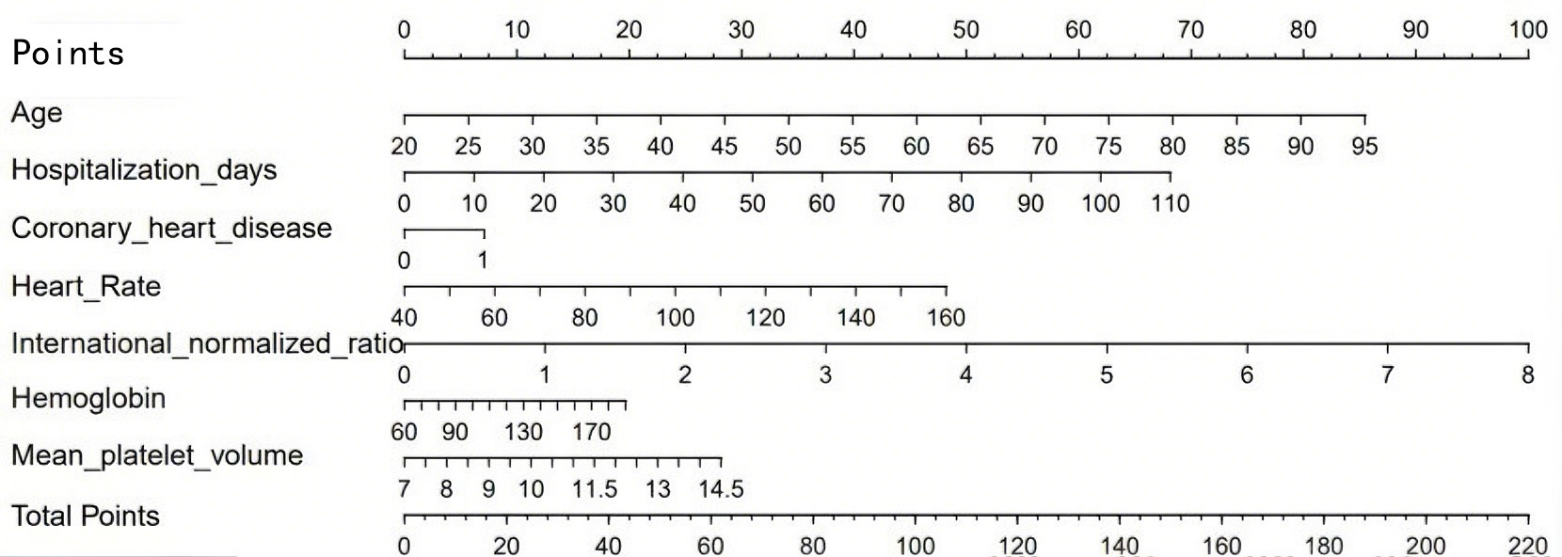
**A nomogram prediction model for AF in AMI patients**. AF, atrial fibrillation; AMI, acute myocardial infarction.

We constructed a nomogram using the independent predictors identified by multivariable logistic regression: age, length of stay, history of coronary heart disease, heart rate, INR, Hb, and mean platelet volume (MPV). The scale (0–100 points) at the top of the graph can measure the score of each variable under the corresponding circumstances. Add the scores of all patient variables corresponding to the scale to form the total score, and correspond to the total score scale and incidence probability at the bottom of the figure, to obtain the predicted risk of AF in patients with AMI (Fig. 2).

### 3.4 Evaluation of Prediction Models

**Fig. 3.**
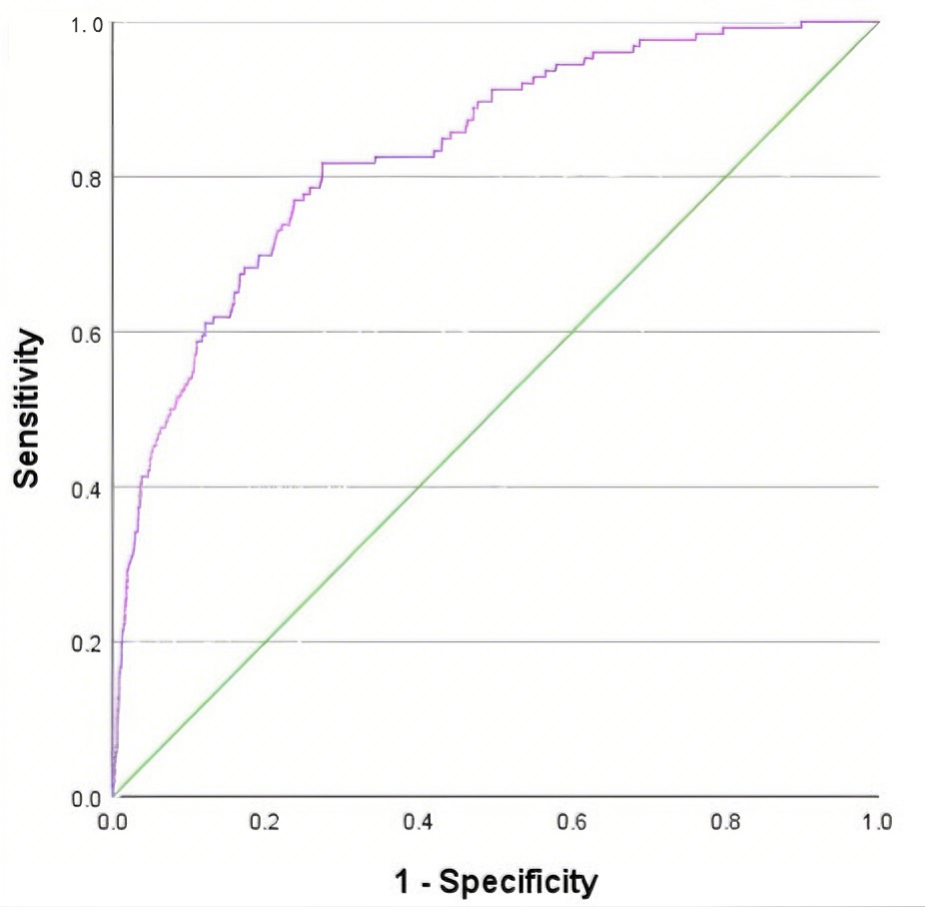
**ROC curve of risk prediction model for AF in AMI patients**.

The predictive effect of nomogram model was analyzed. The results showed that “AUCs” of variables age, hospitalization days, coronary heart disease history, heart rate, INR, Hb, and MPV were 0.721, 0.663, 0.577, 0.614, 0.688, 0.438, and 0.607. The area under the ROC curve AUC of nomogram model for predicting AF in AMI patients was 0.833 (95% *CI*: 0.796–0.870, *p* < 0.001), which had a good predictive effect. The best cutoff value was 0.543, the sensitivity was 0.817, and the specificity was 0.726 (Fig. 3).

**Fig. 4.**
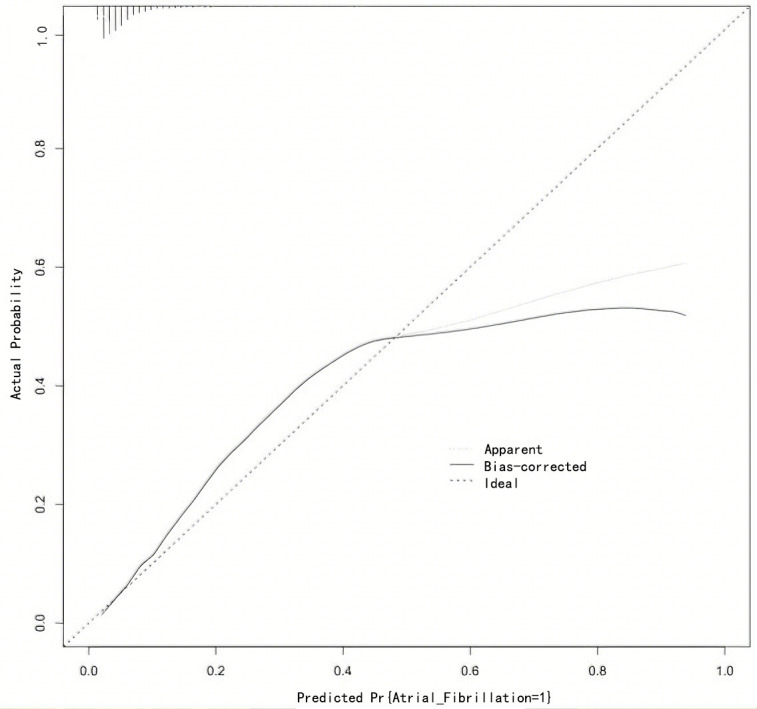
**Calibration curve for Nomogram model**.

**Fig. 5.**
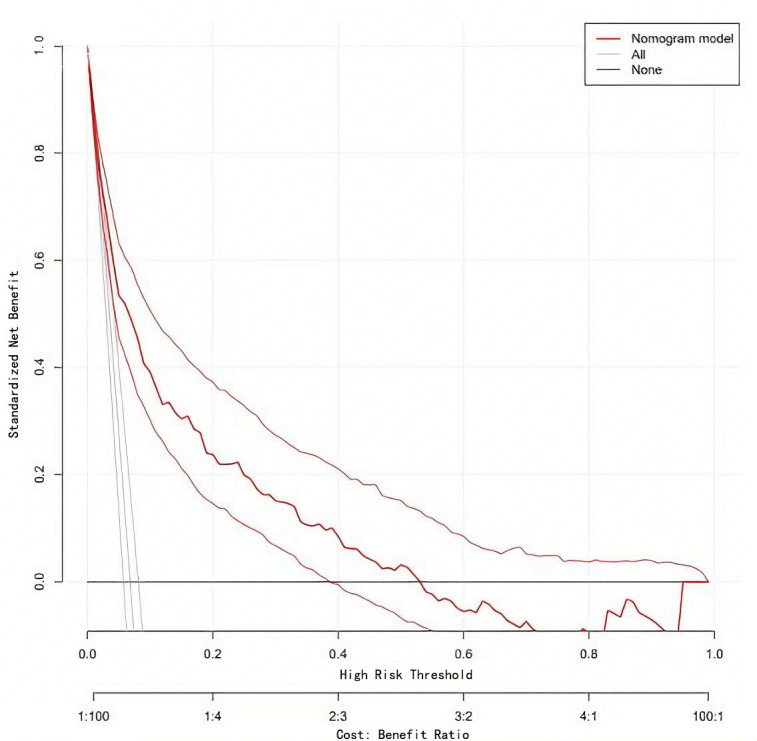
**Decision curve of Nomogram model**.

On the basis of the above, the nomogram model was calibrated and a calibration curve was drawn. The results showed that the predicted values were in good agreement with the measured values (Fig. 4). At the same time, the DCA curve was drawn to calculate the clinical net benefit. DCA indicated that the nomogram model provided a clinical net benefit when the predicted risk threshold for AF exceeded 0.06 (Fig. 5).

## 4. Discussion

This study used multivariate Logistic regression analysis and nomogram model construction to systematically reveal independent risk factors for AF in AMI patients and verify the clinical efficacy of the joint prediction model. The results showed that age, hospitalization days, coronary heart disease history, heart rate, INR, Hb and MPV were independent predictors of AF in AMI patients, and the AUC of the nomogram model was significantly better than that of a single indicator, indicating that the integration of multi-dimensional variables can significantly improve the prediction accuracy. This nomogram model provides a scientific basis for stratification of the risk of AF in AMI patients, and highlights the multi-dimensional effects of underlying disease burden, inflammation-immunity, and myocardial remodeling.

Age is an independent risk factor for AF in AMI patients, and its mechanism involves multiple interactive effects of myocardial aging, atrial fibrosis and autonomic nervous dysfunction [23]. Previous studies have shown that the proportion of patients with AMI complicated by AF is as high as 6%–20%, and the proportion is higher in elderly patients, which is consistent with the results of this study [24]. AF is linked to higher mortality in elderly patients, including those undergoing hip fracture surgery—even without heart failure—and in pacemaker recipients, where it predicts adverse cardiac outcomes [25, 26, 27]. These findings highlight AF as a significant risk factor, underscoring the importance of early identification in AMI patients. It is worth noting that the effect of age on AF in AMI patients may have a non-linear relationship: on the one hand, elderly patients are more prone to electrophysiological disorders due to decreased myocardial elasticity and conduction system degradation [28]; on the other hand, young patients may also be at high risk for AF if they experience severe myocardial damage or genetic atrial matrix abnormalities [29]. The extension of hospitalization days reflects the complexity of the disease and the heterogeneity of treatment response. For example, long-term hospitalizations may induce AF due to repeated myocardial ischemia, electrolyte disorders, or drug side effects such as digitalis toxicity [30]. In addition, extended hospitalization days may also indicate lung infection, deterioration of organ function [31]. Therefore, the number of days in hospital is not only a clinical indicator, but also a “time window” for the dynamic evolution of the disease, which needs to be comprehensively evaluated in conjunction with other biomarkers.

History of coronary heart disease is an independent risk factor for AF in AMI patients, and its mechanism may be related to myocardial scar formation and atrial electrical remodeling [32]. In the past, patients with coronary heart disease were prone to atrial matrix fibrosis and abnormal electrical activity due to multiple myocardial ischemic events, providing a substrate for the development of AF [33]. In addition, coronary heart disease is often accompanied by autonomic dysfunction (such as excessive sympathetic nerve activation), which may promote the development of AF by increasing the heterogeneity of atrial refractory [34]. A fast heart rate suggests excessive activation of the sympathetic-adrenal axis, which not only increases myocardial oxygen consumption, but may also induce reentrant AF by shortening the effective atrial refractory period [35]. It is worth noting that heart rate is very important in the management of AF in patients with AMI: beta blockers can effectively reduce heart rate and improve atrial electrical stability, but the risks of hypotension and deterioration of cardiac function should be vigilant [36]. Therefore, as a predictor of AF in AMI patients, heart rate has clinical significance not only in risk stratification, but also in guiding the optimization of treatment strategies.

Higher INR level suggests the possibility of coagulation dysfunction, which promotes the occurrence of AF through two ways: first, insufficient anticoagulation treatment under high INR conditions is easy to form intraatrial thrombus, leading to local hemodynamic disorders [37]; second, elevated INR is often accompanied by abnormal liver function or vitamin K deficiency, which may indirectly induce AF by interfering with the calcium ion channel function of cardiomyocytes [38]. The mechanisms of the impact of low Hb levels on AF include: insufficient oxygen supply leads to aggravation of myocardial ischemia, which in turn leads to abnormal atrial electrical activity [39]; anemic cardiomyopathy causes ventricular remodeling, which indirectly promotes the occurrence of AF [40]. Therefore, INR and Hb are not only independent predictors of AF in AMI patients, but also important basis for intervention targets (such as optimizing anticoagulation regimens and correcting anemia).

MPV suggests a synergistic effect between platelet activation and inflammatory response [41]. Previous studies have shown that platelet activation can promote atrial thrombosis and aggravate electrophysiological disorders by releasing adenosine diphosphate (ADP) and thromboxane A2 (TXA2) [42]; in addition, elevated MPV may also reflect systemic inflammatory conditions (such as elevated C-reactive protein), which is highly related to the pathological mechanism of AF in AMI patients [43]. It is worth noting that dynamic monitoring of MPV may provide new ideas for early warning of AF in AMI patients: the rapid increase in MPV during the acute phase of AMI may indicate that the inflammatory response is out of control and intensive anti-inflammatory treatment is needed; while the continuous increase in MPV during the recovery phase may impaired myocardial repair and require prolonged anti-platelet therapy [44]. Therefore, MPV is not only a predictor of AF in AMI patients, but also a biomarker reflecting inflammation-thrombosis interaction.

This study built a nomogram prediction model for the risk of AF in AMI patients by integrating multi-dimensional indicators such as age, hospital stay, coronary heart disease history, heart rate, INR, Hb and MPV, providing a new evidence-based tool for clinical practice. The nomogram model (AUC = 0.833) constructed in this study has high practical value in clinical practice. Its advantages are: The selection of variables takes into account the underlying disease burden, inflammation-immune status and clinical dynamic indicators, covering multi-dimensional pathological mechanisms; All variables are clinical routine detection indicators and do not require additional equipment or complex detection processes, and have high clinical practicality; The sensitivity and specificity of the model are good, which can provide a reliable risk threshold in clinical decision-making; Decision curve analysis shows that the model has good clinical net benefits.

Our nomogram provides an interpretable tool for predicting in-hospital AF in AMI. However, machine learning (ML) offers complementary potential by capturing complex patterns in clinical and electrophysiological data. Recent studies demonstrate ML’s value—using P, QRS, and T wave features to predict obstructive coronary disease during treadmill testing, and deep learning models to forecast short-term mortality in pulmonary embolism—highlighting its growing role in cardiovascular risk prediction. Integrating such advanced methods could enhance predictive accuracy in future AF risk models [45, 46]. Future research needs to combine a multidisciplinary perspective to further explore the molecular mechanisms and intervention strategies of AF in AMI patients, and promote the dynamic and individualized development of risk stratification of AF in AMI patients. For example, single-cell sequencing technology is used to analyze the interactions of platelets, lymphocytes and other immune cells in the atrial matrix; machine learning algorithms are used to develop a comprehensive prediction model including biomarkers, imaging features and clinical variables; multi-genomic data (such as genomics, proteomics) and artificial intelligence algorithms are combined to build a more accurate prediction model and explore new intervention strategies for AMI patients with AF (such as anti-inflammatory therapy, atrial electrophysiological regulation). In addition, dynamic monitoring of the changing trends of INR, Hb and MPV may provide more accurate time window information for the prognosis of AMI patients with AF. Eventually, the predictive model of AF in AMI patients’ needs to be integrated with clinical pathways to promote the in-depth application of precision medicine in the field of cardiovascular emergencies.

However, the limitations of the model still need to be addressed: The sample size is relatively small, and its extrapolation needs to be verified through a multi-center prospective study; It does not distinguish between paroxysmal and persistent AF, or between new-onset and pre-existing AF; It does not include joint analysis of imaging indicators (such as left atrial enlargement and abnormal ventricular wall motion), which may underestimate the comprehensiveness of the model; The lack of analysis of the interaction between inflammatory pathways and gene polymorphisms limits the exploration of individualized treatment targets. We explicitly acknowledge potential detection bias arising from variable telemetry exposure vs intermittent ECGs and note that single-center practices may limit generalizability.

## 5. Conclusions

Multivariable prediction model based on age, hospital stay, coronary heart disease history, heart rate, INR, hemoglobin, and average platelet volume has good prediction effect. The variables in this nomogram model are easily obtained in clinical practice and can provide reference for individualized prediction of AF in AMI patients.

## Data Availability

The datasets used and analyzed during the current study are available from the corresponding author on reasonable request.

## Abbreviations

AMI, acute myocardial infarction; AF, atrial fibrillation; PCI, percutaneous coronary intervention; CABG, coronary artery bypass grafting; BMI, body mass index; TG, triglyceride; TC, total cholesterol; HDL-C, high-density lipoprotein cholesterol; LDL-C, low-density lipoprotein cholesterol; APTT, Activated Partial Thromboplastin Time; INR, International Standardized Ratio; FDP, fibrin degradation products; DD, D-dimer; WBC, white blood cells; HB, Hemoglobin; PLT, platelets; MPV, Mean Platelet Volume; NEUT%, Neutrophils Percentage; NEUT, Neutrophils Count; SD, standard deviation; ROC, receiver operating characteristic; DCA, Decision Curve Analysis; ADP, adenosine diphosphate; TXA2, thromboxane A2.
